# Transapical Mitral Valve Implantation After Closure of Posterior Mitral Annulus Cavity With a Nitinol Occluder

**DOI:** 10.1016/j.jaccas.2024.103086

**Published:** 2025-03-05

**Authors:** Annette Maznyczka, Fabien Praz, Nicolas Brugger, Jonas Lanz, Jakub Wiśniewski, David Reineke, Domenico Angellotti, Selim Mosbahi

**Affiliations:** a Department of Cardiology, Inselspital, Bern University Hospital, University of Bern, Bern, Switzerland; b Department of Medicine, Burgdorf Hospital, Burgdorf, Switzerland; c Department of Cardiac Surgery, Inselspital, Bern University Hospital, University of Bern, Bern, Switzerland; d Department of Advanced Biomedical Sciences, University of Naples Federico II, Naples, Italy

**Keywords:** abscess, infective endocarditis, mitral regurgitation, transcatheter mitral valve implantation

## Abstract

Mitral valve infective endocarditis involves a mitral annular abscess in approximately 15% of cases, and surgery is the treatment of choice for this scenario. An 80-year-old comorbid man with eradicated infective endocarditis had an emptied abscess cavity of the posterior mitral valve annulus (with left ventricular–left atrial connections) and had severe paravalvular and coexistent relevant transvalvular mitral regurgitation. He was not a surgical candidate. Successful management was with cavity closure, using a Nitinol occluder device, and transcatheter mitral valve implantation. This case demonstrates a novel transcatheter management approach to treat coexistent postendocarditis paravalvular and relevant transvalvular mitral regurgitation in a patient whose surgical risk was prohibitively high because of comorbidities.

## Visual Summary

**Figure:**
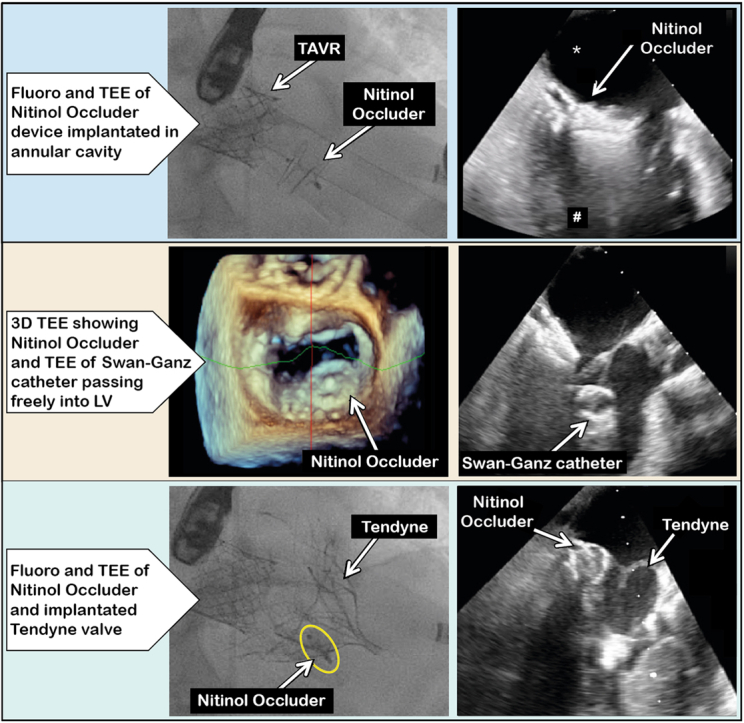

**Visual Summary:**
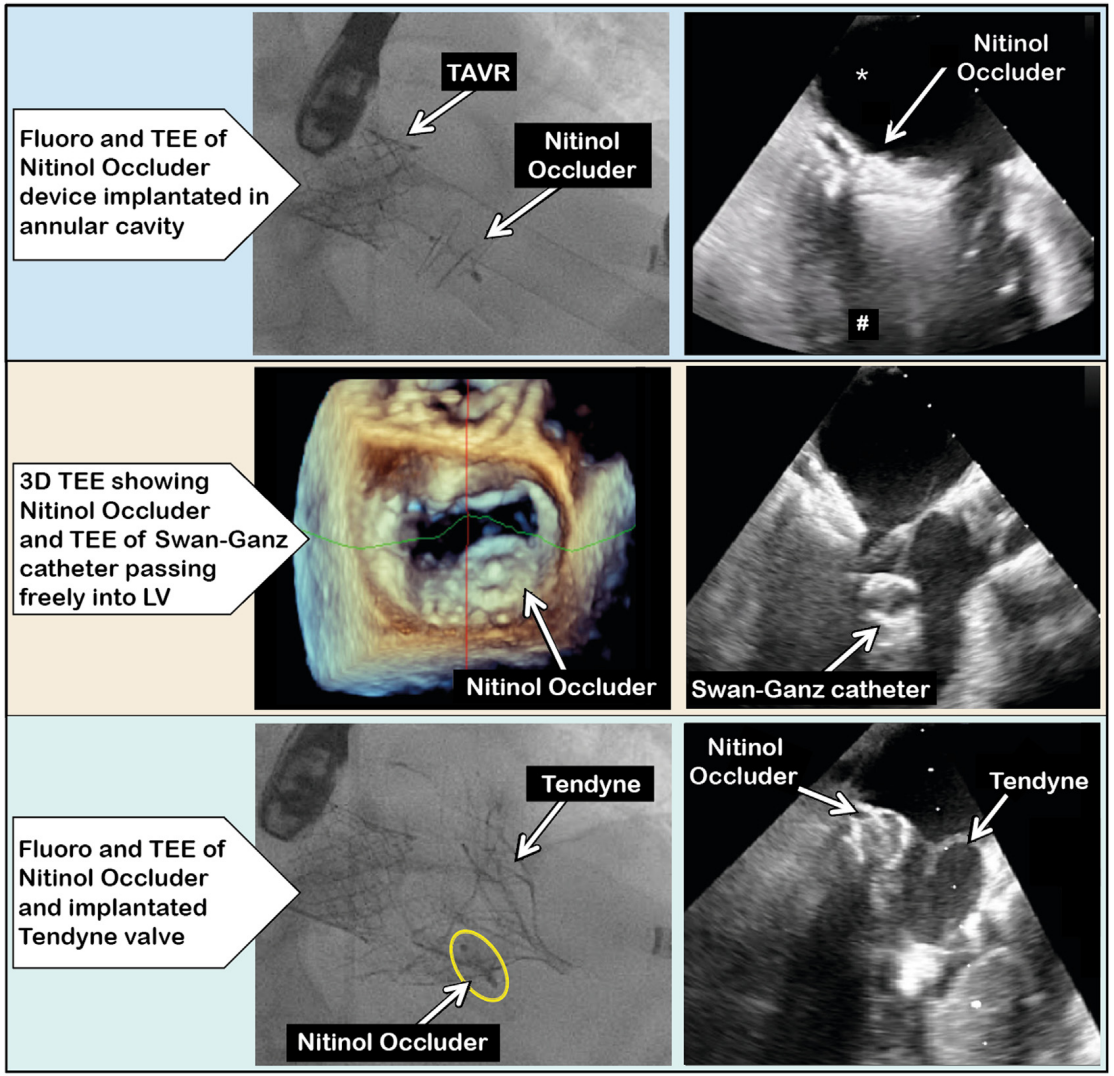
Combined Occluder Device and Transcatheter Mitral Valve Implantation Procedure Asterisk denotes inflow in left atrium and # outflow in left ventricle. LV = left ventricle; TAVR = transcatheter aortic valve replacement; TEE = transesophageal echocardiography.

## History of Presentation

An 80-year-old comorbid man developed bacteremia (staphylococcal and streptococcal) caused by distal phalanx osteomyelitis in his foot. Following amputation, he received ciprofloxacin and rifampicin. Three weeks later, he was rehospitalized with chills, anemia, and dyspnea. The physical examination was notable for a pansystolic murmur, there were no peripheral stigmata of infective endocarditis, and the remainder of the physical examination was unremarkable.

**Take-Home Message**

• This case demonstrates a transcatheter management approach for a cavity in the posterior mitral valve annulus (with LV and left atrial connections), resulting in severe paravalvular mitral regurgitation and coexistent transvalvular mitral regurgitation after eradication of infective endocarditis.

## Past Medical History

The patient had diabetes with peripheral artery disease and paroxysmal atrial fibrillation, and had undergone transcatheter aortic valve replacement 6 years previously for aortic stenosis.

## Differential Diagnosis

The differential diagnosis included infective endocarditis, COVID-19, and pulmonary embolism.

## Investigations

**Figure 1:**
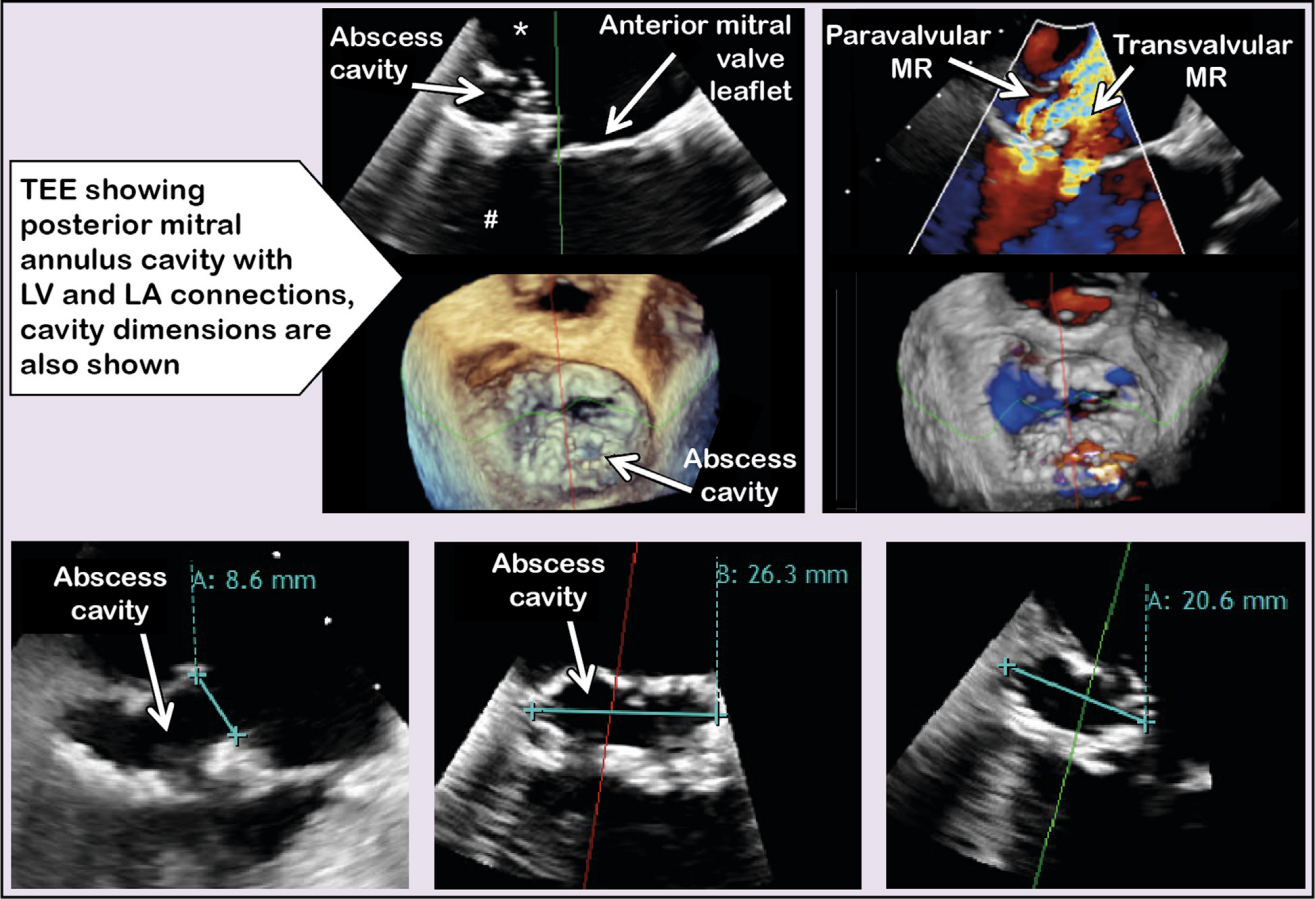
TEE Before Mitral Valve Intervention Showing Transvalvular and Paravalvular MR and a Posterior Mitral Annular Cavity (Dimensions Shown) With LV and LA Connections Asterisk denotes inflow in the left atrium (LA), and pound sign denotes outflow in the left ventricle (LV). MR = mitral regurgitation; TEE = transesophageal echocardiography.

**Figure 2:**
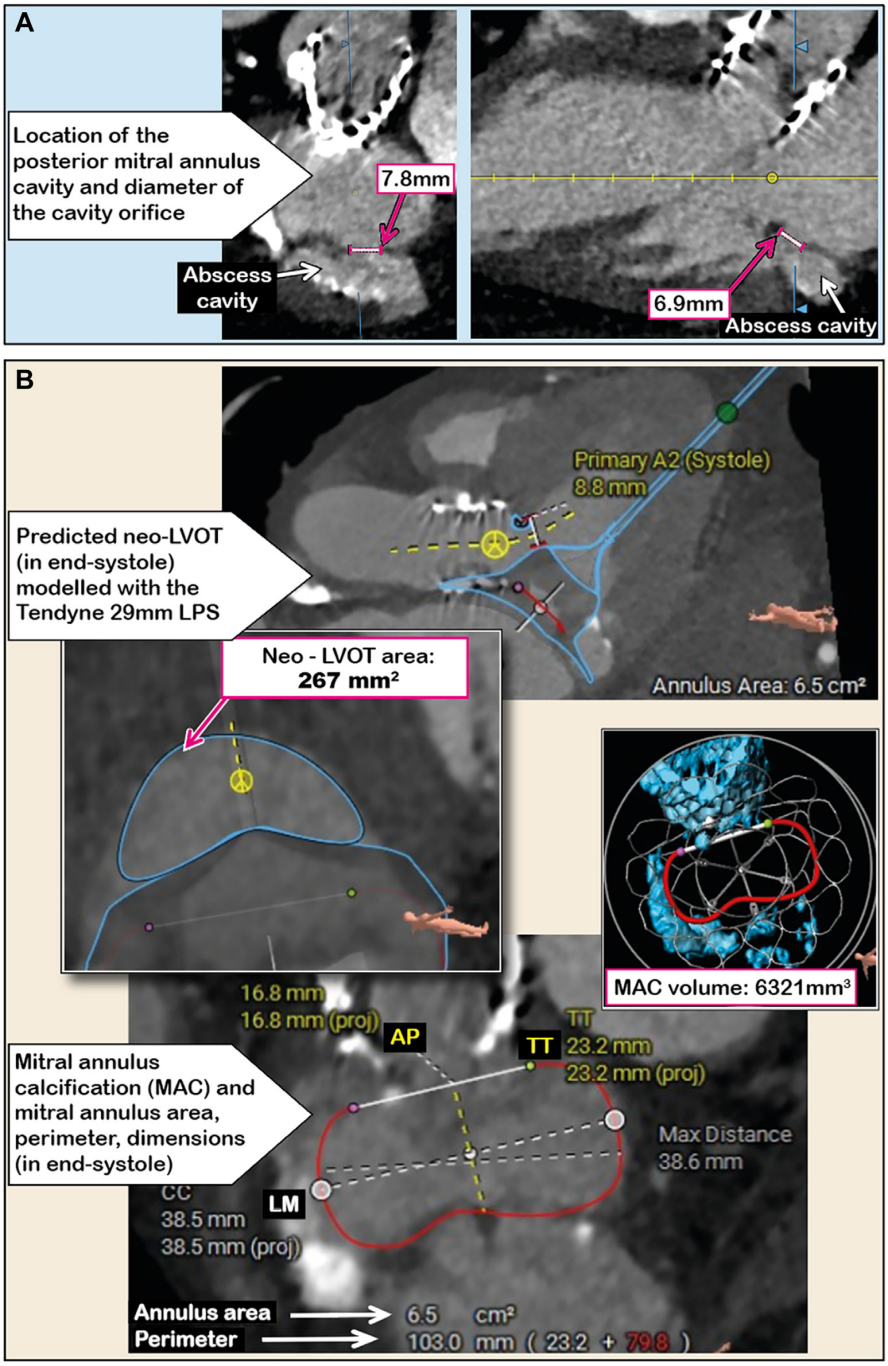
Pre-Procedure Computed Tomography Imaging (A) Positron emission computed tomography performed 1 month preprocedure, showing the location of the posterior mitral annular abscess cavity and diameter of the cavity orifice. (B) Preprocedural multislice computed tomographic evaluation in the end-systolic cardiac phase. Neo–left ventricular outflow tract (LVOT) area was 267 mm^2^; mitral annular anteroposterior (AP) diameter excluding the cavity was 16.8 mm; lateral-medial (LM) diameter was 38.5 mm; intertrigonal (TT) distance was 23.3 mm, area 6.5 cm^2^, and perimeter 103 mm; and moderate mitral annular calcification (MAC) was present (volume 6,321 mm^3^). LPS = low profile system.

Infective endocarditis was confirmed on transesophageal echocardiography, which revealed an emptied abscess cavity of the posterior mitral valve annulus (Figure 1, Video 1), communicating with both the left ventricle (LV) and atrium, resulting in severe paravalvular mitral regurgitation (Video 2) and coexistent relevant transvalvular mitral regurgitation (Video 2). The bioprosthetic aortic valve was functioning well and was not involved in the infective endocarditis. LV function and dimensions were normal. Positron emission computed tomography (Figure 2) characterized the abscess location and dimensions (26 × 19 × 18 mm) and revealed no increased valve enhancement, consistent with serial negative blood cultures, thereby confirming that the infective endocarditis had been eradicated.

## Management

Although surgery is the treatment of choice,^1^^,^^2^ surgical risk was prohibitively high in this case because of frailty, diabetes, peripheral vascular disease, and thrombocytopenia. Therefore, transcatheter mitral valve implantation (TMVI) was considered, in the context of healed infective endocarditis (diagnosed 2 months previously). Preprocedural multislice computed tomographic evaluation included mitral annular dimensions, mitral annular calcification, anterior mitral valve leaflet length, and predicted neo–LV outflow tract (LVOT) area (Figure 2). The data were used to select the size of the Tendyne (Abbott Vascular) TMVI device. During TMVI, the anterior mitral valve leaflet is displaced toward the LVOT, creating a confined neo-LVOT area, also restricted by the covered section of the prosthetic valve frame and the systolic thickening of the basal LV septum. Particularly unpredictable is the occurrence of systolic anterior motion of the tip of the anterior mitral valve leaflet, which can be aspirated into the LVOT. In our case, the end-systolic neo-LVOT was 267 mm^2^ when modeled with a Tendyne 29 mm low profile bioprosthesis, indicating low risk for LVOT obstruction^3^ (Figure 2).

Incomplete seal of the paravalvular mitral regurgitation by the TMVI device was anticipated, so we planned to at least partially close the cavity connections using a Nitinol patent foramen ovale (PFO) occluder device. The final decision regarding the Nitinol occluder size was made on the day of the procedure, on the basis of the intraprocedural 3-dimensional transesophageal echocardiographic images, which showed an abscess cavity orifice of 8.6 mm, for a total longitudinal size of about 20 to 26 mm (Figure 1), which had relevantly increased compared with computed tomography performed 1 month earlier (Figure 2). An 18-mm Nitinol PFO occluder was therefore selected on the basis of its small profile minimizing interaction with the mitral bioprosthesis and its anticipated ability to collapse the cavity. Anterolateral left thoracotomy was performed, followed by transapical placement of an 18-mm Amplatzer Talisman PFO occluder (Abbott Vascular) with the distal disc in the left atrium and the proximal one under the mitral valve in the LV, reducing the paravalvular regurgitation to moderate (Video 3). TMVI was then performed to treat the residual para- and transvalvular mitral regurgitation. Correct transmitral wire position was confirmed on transesophageal echocardiography, and smooth passage of an inflated Swan-Ganz catheter indicated no chordae entanglement. The Tendyne delivery system was inserted into the left atrium, and the Nitinol occluder device position remained stable when the balloon dilator of the Tendyne delivery catheter was inserted (Video 4). A transapical Tendyne 29-mm low profile mitral valve prosthesis was implanted, leaving only trace regurgitation at the interface between the occluder and the prosthetic valve (Video 5). The 3-dimensional transesophageal echocardiographic images after deployment of the Tendyne device are shown in Video 6. The postprocedural peak LVOT gradient was 20 mm Hg, and the mean mitral valve gradient was 5 mm Hg. Apixaban was started 2 days later.

## Discussion

Approximately 15% of cases of mitral valve infective endocarditis involve a mitral annular abscess, typically located in the posterior mitral annulus.^4^ Surgical treatment mandates careful debridement and reconstruction of the atrioventricular groove with a patch, prior to mitral valve replacement.^4^ In our case, a transcatheter approach could be considered because the infection had been eradicated, and therefore tissue debridement was not required before TMVI.

In this case, Tendyne TMVI was selected since it is the only mitral transcatheter replacement system approved in Europe. The Tendyne device is an intra-annular trileaflet porcine pericardial valve, with an inner circular self-expanding Nitinol stent and outer D-shaped self-expanding Nitinol stent. The outer frame has a supra-annular cuff, which provides atrial sealing and anchoring and minimizes paravalvular mitral regurgitation. It is anchored to the LV apex by an epicardial pad tether. Transapical and transseptal are the 2 principal access routes used for TMVI. Even though the transseptal approach is less invasive, it causes a large atrial septal defect and is technically challenging because of the small operating space.^5^ The transapical approach enables precise perpendicular positioning and anchoring of the valve prosthesis, but bleeding and myocardial injury are potential risks.

We decided to use a Nitinol PFO occluder to seal the abscess cavity by apposing the ventricular and atrial surfaces of the cavity, prior to TMVI. The Nitinol PFO occluder is a double-disc device with a short waist and small profile and therefore achieved the desired result of collapsing the cavity space. A more bulky device with a “body” that would fill the healed abscess cavity could theoretically have resulted in distortion of the Tendyne device, interference with the anchoring and sealing of the TMVI, or anterior dislodgement increasing the risk of LVOT obstruction. The fact that the paravalvular mitral regurgitation was reduced after the Nitinol occluder device was implanted suggests that sealing of the abscess cavity was contributed to by the Nitinol occluder device, not solely by the atrial cuff of the Tendyne device.

## Follow-Up

**Figure 3:**
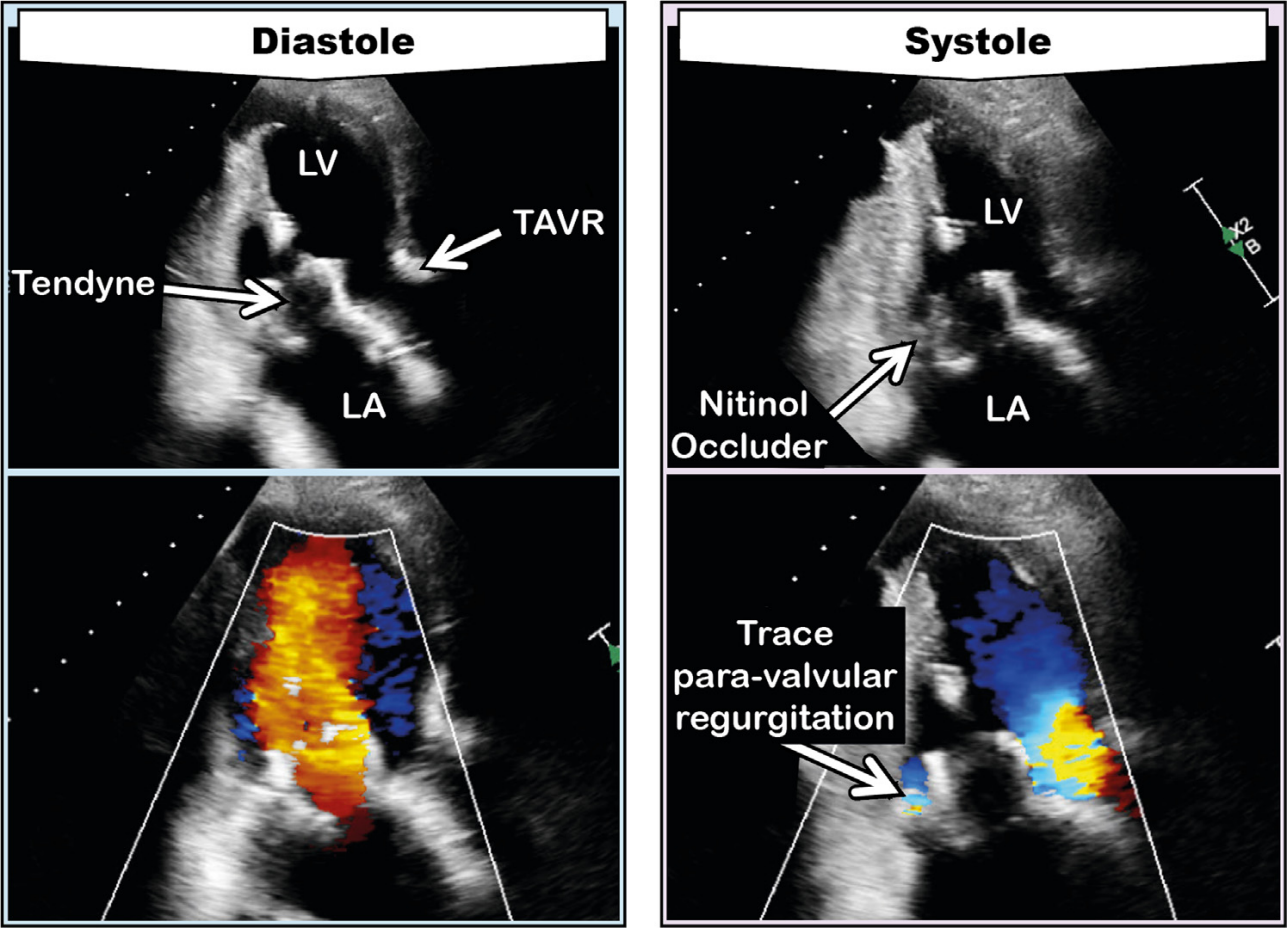
Transthoracic Echocardiogram 3 Months Postprocedure Showing Trace Paravalvular Mitral Regurgitation Abbreviations as in Figure 1 and the Visual Summary.

At 3-month cardiology outpatient follow-up, the patient’s dyspnea had diminished, and transthoracic echocardiography showed no transvalvular mitral regurgitation and only trace paravalvular mitral regurgitation (Figure 3).

## Conclusions

This case demonstrates a first-in-human procedure to treat coexistent postendocarditis large paravalvular and relevant transvalvular mitral regurgitation, using a Nitinol PFO occluder device to close a posterior annulus cavity, followed by TMVI.

## Funding Support and Author Disclosures

Dr Maznyczka is a European Association of Percutaneous Cardiovascular Interventions international structural fellow (funded by Edwards Lifesciences through the European Association of Percutaneous Cardiovascular Interventions and not directly by Edwards Lifesciences); and has received travel expenses from Edwards Lifesciences, Abbott Laboratories, Boston Scientific, and Medtronic. Dr Praz has received travel expenses from Abbott Vascular, Edwards Lifesciences, Medira, Siemens Healthineers, and InQB8 Medical Technologies; and has received a research grant to the institution from Abbott Vascular. Dr Lanz has received speaker fees to the institution from Edwards Lifesciences and Abbott. Dr Reineke has received travel expenses from Abbott Laboratories, Edwards Lifesciences, and Medtronic; and has proctoring and consulting contracts with Abbott Laboratories and Medtronic. Dr Angellotti is supported by a research grant provided by the CardioPath PhD program. All other authors have reported that they have no relationships relevant to the contents of this paper to disclose.

## Appendix

Video 1 Transesophageal Echocardiography Showing Posterior Mitral Annular Cavity

Video 2 Transesophageal Echocardiography With Color Flow Doppler Showing Mitral Regurgitation

Video 3 Transesophageal Echocardiography Showing Mitral Regurgitation After Implantation of the Nitinol Occluder Device

Video 4 Fluoroscopy Showing Insertion of Balloon Dilator at the Tip of the Tendyne Delivery Catheter

Video 5 Transesophageal echocardiography Postprocedure After Tendyne Implantation

Video 6 Three-Dimensional Transesophageal Echocardiography Postprocedure After Tendyne Implantation
